# Fatty Acid Ethyl Esters (FAEE): A New, Green and Renewable Solvent for the Extraction of Carotenoids from Tomato Waste Products

**DOI:** 10.3390/molecules26144388

**Published:** 2021-07-20

**Authors:** Aurel Diacon, Ioan Călinescu, Mircea Vinatoru, Petre Chipurici, Alexandru Vlaicu, Aurelian Cristian Boscornea, Timothy J. Mason

**Affiliations:** 1 Bioresources and Polymer Science Department, Faculty of Applied Chemistry and Materials Science, University Politehnica of Bucharest, 1-7 Gh. Polizu Str., 011061 Bucharest, Romania; aurel.diacon@upb.ro (A.D.); ioan.calinescu@upb.ro (I.C.); mircea.vinatoru@upb.ro (M.V.); alexandru.vlaicu@icechim.ro (A.V.); cristian.boscornea@upb.ro (A.C.B.); 2 National Research & Development Institute for Chemistry and Petrochemistry ICECHIM, 202 Splaiul Independenței St., 060021 Bucharest, Romania; 3 Faculty of Health and Life Sciences, Coventry University, Priory Street, Coventry CV1 5FB, UK; t.mason@coventry.ac.uk

**Keywords:** FAEE, green solvent, green technique, beta-carotene, lycopene, ultrasound-assisted extraction (UAE), UV-vis spectroscopy

## Abstract

Currently there is a drive towards the minimisation and reclamation of valuable materials from the waste products of the food and beverage industry. This can be achieved through the extraction of residual nutraceuticals from such materials. Tomato pomace contains carotenoids and other chemicals which can be extracted directly into edible oils to improve the health-giving properties of such oils. We report here a novel green solvent, fatty acid ethyl esters (FAEE), which is significantly more effective than sunflower oil and hexane for the extraction of lycopene and beta-carotene from tomato skin waste. FAEE are a non-toxic renewable resource that is environmentally friendly and to our knowledge has never been used as a vegetal extraction fluid. The efficiency of FAEE extraction was significantly improved relative to both sunflower oil and hexane under ultrasound-assisted extraction (UAE) conditions. In addition, FAEE have the additional and significant advantage that once enriched with the extracted nutraceuticals can be used directly as a food additive.

## 1. Introduction

Green Chemistry is built on five basic concepts: (i) prevention, (ii) the better use of raw materials, (iii) better waste management, (iv) energy savings, and (v) the use of solvents that are compatible with the environment [1]. Sonochemistry has been linked with green chemistry for many years because of the ways in which it can assist with these concepts [2].

The last of these concepts, the use of green solvents, has been questioned in recent years. Cintas has pointed out that simply accelerating a reaction with the use of smaller amounts of solvent or, in the case of microwave chemistry, the use of no solvent at all may not be truly green [3]. He argues that such processes may not be green when the work-up protocol is taken into account where product isolation and purification may well require liquid-liquid extraction followed by chromatography with large volumes of volatile organic solvents (VOCs). Here, the final product isolation and purification may require extensive uses of organic solvent for liquid-liquid extraction and chromatography.

Ultrasound-assisted extraction (UAE) is important for the improved extraction of materials from vegetal resources because it affords improved and more effective extraction. This can lead to improvements in the yield of heat-sensitive components which suffer degradation under prolonged conventional extraction [4,5]. There is no doubt that such a technology does afford a more efficient process, but when common solvents such as hexane or ethyl acetate are employed the UAE cannot be claimed to be fully green because both are petroleum-based.

There are some examples of the use of more environmentally-friendly solvents in the UAE. One example involves the improvement in the nutritional value of a low-quality edible oil such as non-virgin olive oil. The conventional methodology involves a two-stage process where nutraceutical compounds obtained separately from the solvent extraction of plant resources are added to the olive oil. A much greener approach is to use the edible oil itself as the extraction solvent. Thus, UAE has been used to enrich low-grade olive oils with nutraceuticals (carotenoids) from the waste material remaining after sea buckthorn extraction [6]. Tomato is also a rich source of carotenoids, and tomato processing generates from 3% to 7% (by weight) of waste. This pomace contains lycopene together with phenolics, organic acids, fibres, and many other components of nutritional benefit [7,8,9]. Currently, ethyl acetate, not regarded as a green solvent, is used for carotenoid extraction from this material [10,11].

Research efforts have been directed towards the development of new green solvents [12,13]. One example of this is ethyl lactate, produced by fermentation from biomass [14,15]. We have identified another promising candidate which is fatty acid ethyl esters (FAEE). This material is an important constituent of biodiesel. In our laboratories, we have found it to be an excellent solvent for some vegetal extractions and it has very strong green credentials such as being renewable from natural raw materials, environmentally friendly, and non-toxic [16,17,18,19,20]. It has also been shown to have some benefits as a fatty acid food supplement [21]. This beneficial property can be further enhanced by the inclusion of carotenoids derived from tomato pomace. We report here a comparison of carotenoid extraction from tomato waste under conventional and UAE conditions using three solvents: hexane, sunflower oil, and FAEE.

## 2. Experimental

The extraction was carried out using waste skins from the processing of tomatoes (variety *Pontica 102*) which are spherically-shaped red tomatoes, with an average weight of 90–150 g, cultivated in the field in the Naipu-Ghimpati area, Giurgiu district, Romania. The peels were obtained via a conventional kitchen appliance to make tomato juice. The peels were collected and processed as described in Section 2.1.

The solvents used for extraction were fatty acid ethyl esters (FAEE)—obtained by the transesterification of sunflower oil (Carrefour brand, Bucharest, Romania), sunflower oil (Carrefour brand, Bucharest, Romania) itself, and hexane (≥97%, EMD Millipore, Darmstadt, Germany). The lycopene and beta-carotene standards were purchased from Sigma-Aldrich (St. Louis, MO, USA).

The FAEE were obtained by transesterification of sunflower oil using anhydrous ethanol (1/12 molar ratio oil: ethanol) and NaOH as a catalyst (0.17% weight vs. oil) at 50 °C, for a reaction time of 1 h. The reaction mixture was concentrated by vacuum distillation to remove excess ethanol, followed by phase separation to recover the FAEE layer. The FAEE were further purified by vacuum distillation (20 mmHg upper boiling point of 220 °C) to a purity of 98% (determined by GC analysis) [22]. The product was slightly yellow, the acidity index determined was 0.02 mg KOH/g sample [23], the peroxide index was 3.5 (milliequivalents oxygen/kg of FAEE) [24], and the iodine value was 100 g I_2_/100 g of FAEE [25].

The lycopene and beta-carotene concentrations were determined by UV-vis spectroscopy using an Able Jasco V-550 spectrophotometer (Able Jasco, Tokyo, Japan). The determinations involved the recording of the calibration curves for each component in each solvent and using the extinction coefficient of the two components in each solvent. Thus, using a similar strategy and data published by Fish [26] for hexane, three equation sets were determined (Table 1) using the absorbance values of 450 and 503 nm to estimate the concentrations of beta-carotene and lycopene. This involved the measurement of the calibration curves for beta-carotene and lycopene (at the two selected wavelengths) and building an equation set for each solvent as described by Fish [26]. The calibration curves obtained for beta-carotene and lycopene were linear, with an R^2^ value between 0.983 and 0.997, for a concentration of beta-carotene and lycopene between 1 and 20 mg/L.

From the equations in Table 1, we obtained the concentration of beta-carotene and lycopene expressed in mg/L. To transform into mg/100 g of dried plant the next formula must be applied:

$$\frac{\mathrm{mg}}{100\;g\;dry\;plant}=concentration\;from\;UV\;\left(\frac{\mathrm{mg}}{\mathrm{mL}}\right)\times \frac{40\;\left(\mathrm{mL}\;volume\;of\;solvent\;used\right)}{2\;\left(g\;of\;dry\;plant\;used\right)}\times 100$$

The extraction process was monitored by collecting samples at specified intervals from each experiment, diluting the sample with the extraction solvent, and analysing the absorbance spectra.

### 2.1. Pre-Treatment of the Vegetal Material

For a more accurate evaluation of the lycopene and beta-carotene extraction process, the plant material was subjected to a preliminary pre-treatment procedure. The skins were slowly dried (6 days at 45 °C in a forced convection oven, until a constant mass was attained). The drying is necessary to remove the moisture and to allow the extraction in an organic solvent, to make grinding easier. The powdered material was sieved and the fraction under 200 µm was selected for use in the extraction of carotenoids.

### 2.2. Extraction Process Conditions

**Figure 1:**
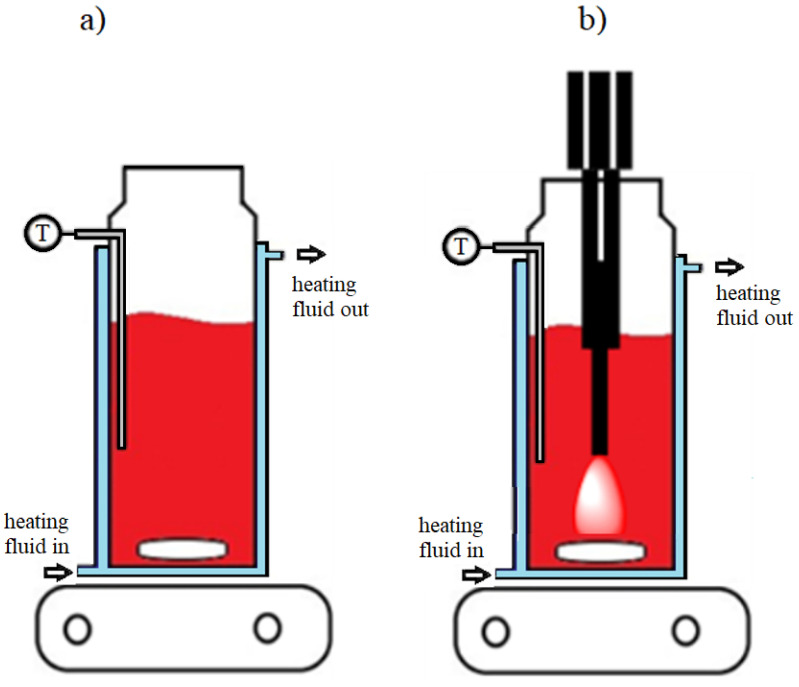
Extraction experiments set-up for (**a**) conventional and (**b**) ultrasound-assisted extractions.

In each extraction experiment, 40 mL of solvent (hexane, sunflower oil, or FAEE) and 2 g of dried and sieved vegetal material was used. The conventional extraction experiments were performed in a glass reactor equipped with a heating mantle, temperature sensor, and magnetic stirrer (800 rpm) (Figure 1a). A constant temperature (60 °C) was maintained throughout the extraction with the aid of heating fluid and a thermostat, and the temperature was monitored using a sensor inserted into the extraction mixture. The solvent was always preheated prior to the addition of the plant material.

The extraction experiments were performed in triplicate to evaluate their reproducibility.

### 2.3. Ultrasonic-Assisted Extraction Process

Figure 1b shows the arrangements for the ultrasound-assisted extractions. UAE was performed using the probe systems (Vibracell 750VCX (Sonics & Materials Inc., Newtown, CT, USA)—with a tip diameter of 13 mm), and the energy delivered by the probe system was calorimetrically determined [27,28]. The ultrasonic device was operated in pulse mode (5 s on and 5 s off) to control the reaction temperature and reduce oxidative degradation of the carotenoids [29]. The powers determined for the ultrasonic input are shown in Table 2.

## 3. Results and Discussions

**Figure 2:**
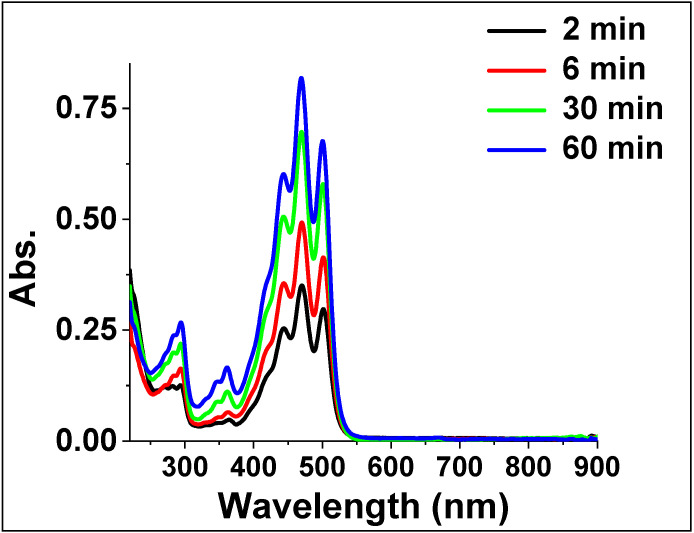
UV-vis absorption spectra of the extracts from the *Pontica 102* skins in hexane, under conventional conditions at 60 °C.

Extraction experiments of the *Pontica 102* tomato skins under conventional conditions were performed with hexane as the solvent, and the resulting UV-visible absorption spectra of the extracts are shown (Figure 2).

**Figure 3:**
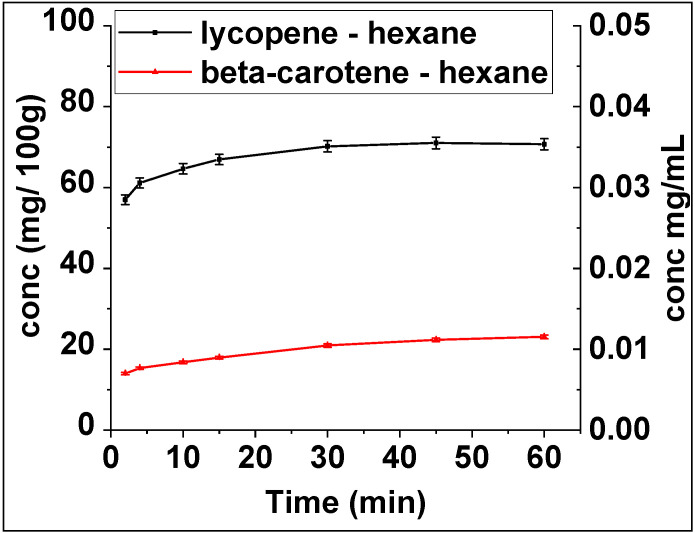
Change in lycopene and beta-carotene concentration (mg carotenoid/100 g dry plant (left y-axis) and mg carotenoid/mL solvent (right y-axis)) during extraction from *Pontica 102* skins with hexane, under conventional conditions at 60 °C.

Further analysis revealed that the concentration of lycopene was higher than beta-carotene in accordance with previously reported results (Figure 3) [30,31,32].

As stated above, hexane is a good choice as a solvent for carotenoids in terms of extraction yield, but it is not green. For this reason, two green solvents were used for comparison purposes: sunflower oil and the fatty acid ethyl esters (FAEE) derived from it. The process of the transesterification of sunflower oil followed by distillation produces FAEE with essentially no contaminants making it ideal for applications involving the extraction of natural materials.

**Figure 4:**
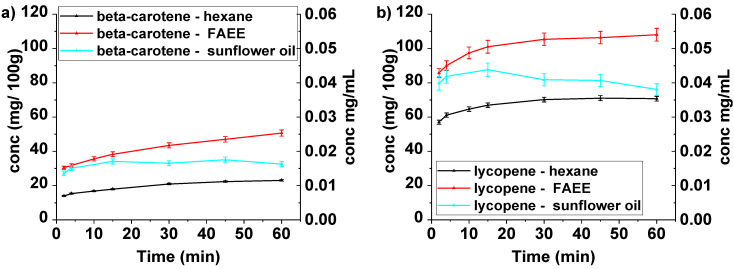
Beta-carotene (**a**) and lycopene (**b**) concentrations (mg carotenoid/100 g dry plant (left y-axis) and mg carotenoid/mL solvent (right y-axis)) during extraction experiments from *Pontica 102* skins in different solvents, under conventional conditions at 60 °C.

**Figure 5:**
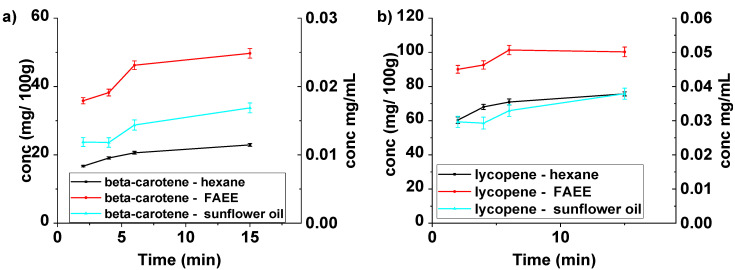
Beta-carotene (**a**) and lycopene (**b**) concentration (mg carotenoid/100 g dry plant (left y-axis) and mg carotenoid/mL solvent (right y-axis)) during extraction experiments from *Pontica 102* skins in different solvents, at 60 °C, under US irradiation using Vibracell VCX 750.

All three solvents were used under conventional conditions (magnetically stirring at 60 °C) (Figure 4) and ultrasound-assisted extraction conditions (Figure 5). These figures clearly show that FAEE is the solvent of choice for both beta-carotene and lycopene. Compared with hexane, the yields after 60 min are improved by factors of 2.2 and 1.5, respectively, under conventional conditions.

The use of ultrasound on these extractions would be expected to lead to improvements in the yields [4] and the results of UAE as shown in Figure 5. In all cases, the UAE was essentially completed after 15 min.

Analysis of the results in Figure 4 and Figure 5 reveals the significant increases in extraction yields obtained using UAE for FAEE extraction compared with conventional extraction. For the FAEE extraction, the concentration of beta-carotene extracted by ultrasound in 15 min is 0.025 mg/mL (49.7 mg/100 g) which is 30% higher than that obtained conventionally after 15 min 0.019 mg/mL (38.3 mg/100 g) and approximately equal to that obtained conventionally after 60 min 0.025 mg/mL (50.6 mg/100 g). In the case of lycopene using the same solvent, the maximum concentration is reached at 6 min 0.051 mg/mL (101.4 mg/100 g) using UAE. This is equivalent to that achieved after 15 min under conventional conditions.

**Figure 6:**
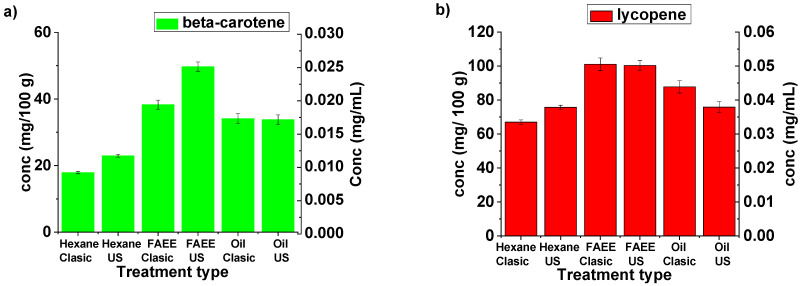
Changes in beta-carotene (**a**) and lycopene (**b**) concentration (mg carotenoid/100 g dry plant (left y-axis) and mg carotenoid/mL solvent (right y-axis)) induced by ultrasound after 15 min.

Changes in the yields of beta-carotene and lycopene induced by UAE are represented differently in Figure 6. Here it can be seen that for both materials FAEE is the best solvent although in the case of lycopene the yield is not improved using UAE but is reached in a significantly shorter time (6 min).

Extraction with hexane was improved by UAE in both cases. The yield of beta-carotene with UAE after 15 min 0.011 mg/mL (22.9 mg/100 g) was significantly higher than the amount extracted conventionally at the same time 0.009 mg/mL (17.9 mg/100 g) and very close to the conventional yield after 60 min 0.012 mg/mL (23.05 mg/100 g). After 15 min under UAE conditions the lycopene yielded 0.038 mg/mL (75.7 mg/100 g) around 10% higher than that obtained conventionally after 60 min 0.035 mg/mL (70.7 mg/100 g). The relative standard deviation values varied between 1.92 and 4.46% for beta-carotene and lycopene.

UAE has little effect on extractions using sunflower oil and this can be ascribed to the fact that sunflower oil is more viscous at the chosen temperature (60 °C) (21.8 mPa·s [33]) than FAEE (0.75 mPa·s [34]) and is, therefore, less prone to cavitation. At the same time, the viscosity of FAEE is low enough to facilitate mass transfer while the viscosity of sunflower oil is still high.

## 4. Conclusions

Two important nutraceuticals, beta-carotene, and lycopene were extracted from the waste products, mainly skin, from the processing of tomatoes (variety *Pontica 102*). The solvents used were hexane, sunflower oil, and fatty acid ethyl esters (FAEE), a bio-based renewable solvent. Compared with hexane extraction, FAEE improved the yields after 60 min by factors of 2.2 and 1.5, respectively. Overall, the order of extraction efficiency under stirred conditions was: FAEE > sunflower oil > hexane.

Using the UAE (ultrasound-assisted extraction) after 15 min (Vibracell VCX 750) at the same temperature, the extraction of beta-carotene with hexane rose from 0.009 to 0.0114 mg/mL (+23%); using the FAEE, the improvement was from 0.020 to 0.025 mg/mL (+30%). For lycopene, the UAE increased the yield in hexane from 0.034 to 0.038 mg/mL (+12%) but with the FAEE there was no increase in the case of lycopene which remained at 0.05 mg/mL but in a much shorter time under the UAE and still substantially more than was obtained using hexane. The UAE had no significant effect on the extractions using sunflower oil.

The use of FAEE, a non-toxic, environmentally-friendly renewable resource, is a better solvent for the extraction of both beta-carotene and lycopene from tomato pomace. To our knowledge, FAEE have never before been used as a vegetal extraction fluid.

## 5. Patents 

Calinescu: I.: Vinatoru, M.; Diacon, A.; Chipurici, P. Provisional Patent Application, Biodegradable and non-toxic solvent for the extraction of liposoluble active principles. Provisional Patent Application RO133507-A0, “Biodegradable and non-toxic solvent for the extraction of liposoluble active principles”, 2018.

## Figures and Tables

**Table 1:** Equations for the UV-vis spectroscopy determination of beta-carotene and lycopene.

Solvent	Beta-Carotene, mg/L	Lycopene, mg/L
Hexane	${\left[\beta -carotene\right]}_{\frac{\mathrm{mg}}{L}}=4.367A_{450}-2.947A_{503}$	${\left[Lycopene\right]}_{\frac{\mathrm{mg}}{L}}=3.521A_{503}-0.587A_{450}$
Sunflower oil	${\left[\beta -carotene\right]}_{\frac{\mathrm{mg}}{L}}=8.871A_{450}-5.987A_{503}$	${\left[Lycopene\right]}_{\frac{\mathrm{mg}}{L}}=7.153A_{503}-1.192A_{450}$
FAEE	${\left[\beta -carotene\right]}_{\frac{\mathrm{mg}}{L}}=9.311A_{450}-6.283A_{503}$	${\left[Lycopene\right]}_{\frac{\mathrm{mg}}{L}}=7.507A_{503}-1.252A_{450}$

**Table 2:** US energy depending on the equipment and extraction solvent (determined at 60 °C).

Equipment	US Power Density W/mL		
	Hexane	FAEE	Sunflower Oil
Vibracell VCX750	0.22	0.29	0.29

## Sample Availability

Samples of the compounds are not available from the authors.
